# Predicting inadequate postoperative pain management in depressed patients: A machine learning approach

**DOI:** 10.1371/journal.pone.0210575

**Published:** 2019-02-06

**Authors:** Arjun Parthipan, Imon Banerjee, Keith Humphreys, Steven M. Asch, Catherine Curtin, Ian Carroll, Tina Hernandez-Boussard

**Affiliations:** 1 Department of Management Science and Engineering, Stanford University, Stanford, California, United States of America; 2 Department of Biomedical Data Sciences, Stanford University, Stanford, California, United States of America; 3 Department of Psychiatry, Stanford University, Stanford, California, United States of America; 4 VA Palo Alto Center for Innovation to Implementation, Palo Alto, California, United States of America; 5 Department of Medicine, Stanford University, Stanford, California, United States of America; 6 Department of Surgery, VA Palo Alto Healthcare System, Palo Alto, California, United States of America; 7 Department of Surgery, Stanford University, Stanford, California, United States of America; 8 Department of Anesthesiology, Stanford University, Stanford, California, United States of America; University of Murcia, SPAIN

## Abstract

Widely-prescribed prodrug opioids (e.g., hydrocodone) require conversion by liver enzyme CYP-2D6 to exert their analgesic effects. The most commonly prescribed antidepressant, selective serotonin reuptake inhibitors (SSRIs), inhibits CYP-2D6 activity and therefore may reduce the effectiveness of prodrug opioids. We used a machine learning approach to identify patients prescribed a combination of SSRIs and prodrug opioids postoperatively and to examine the effect of this combination on postoperative pain control. Using EHR data from an academic medical center, we identified patients receiving surgery over a 9-year period. We developed and validated natural language processing (NLP) algorithms to extract depression-related information (diagnosis, SSRI use, symptoms) from structured and unstructured data elements. The primary outcome was the difference between preoperative pain score and postoperative pain at discharge, 3-week and 8-week time points. We developed computational models to predict the increase or decrease in the postoperative pain across the 3 time points by using the patient’s EHR data (e.g. medications, vitals, demographics) captured before surgery. We evaluate the generalizability of the model using 10-fold cross-validation method where the holdout test method is repeated 10 times and mean area-under-the-curve (AUC) is considered as evaluation metrics for the prediction performance. We identified 4,306 surgical patients with symptoms of depression. A total of 14.1% were prescribed both an SSRI and a prodrug opioid, 29.4% were prescribed an SSRI and a non-prodrug opioid, 18.6% were prescribed a prodrug opioid but were not on SSRIs, and 37.5% were prescribed a non-prodrug opioid but were not on SSRIs. Our NLP algorithm identified depression with a F1 score of 0.95 against manual annotation of 300 randomly sampled clinical notes. On average, patients receiving prodrug opioids had lower average pain scores (p<0.05), with the exception of the SSRI+ group at 3-weeks postoperative follow-up. However, SSRI+/Prodrug+ had significantly worse pain control at discharge, 3 and 8-week follow-up (p < .01) compared to SSRI+/Prodrug- patients, whereas there was no difference in pain control among the SSRI- patients by prodrug opioid (p>0.05). The machine learning algorithm accurately predicted the increase or decrease of the discharge, 3-week and 8-week follow-up pain scores when compared to the pre-operative pain score using 10-fold cross validation (mean area under the receiver operating characteristic curve 0.87, 0.81, and 0.69, respectively). Preoperative pain, surgery type, and opioid tolerance were the strongest predictors of postoperative pain control. We provide the first direct clinical evidence that the known ability of SSRIs to inhibit prodrug opioid effectiveness is associated with worse pain control among depressed patients. Current prescribing patterns indicate that prescribers may not account for this interaction when choosing an opioid. The study results imply that prescribers might instead choose direct acting opioids (e.g. oxycodone or morphine) in depressed patients on SSRIs.

## Introduction

Opioids are currently a first-line treatment of postoperative pain and surgery may be a gateway to opioid misuse [1,2]. Most surgical patients receive opioids, regardless of co-morbidities, prior opioid-related problems, or possible drug-drug interactions [3]. Depression is a common comorbidity and effects postoperative pain management. Longitudinal epidemiologic studies evaluating depression indicate that patients with depression are between two to five times more likely to have a new chronic pain problem at follow-up from one to eight years later [4–6]. Mental illness also increases the risk for opioid prescription abuse [7]. Antidepressants are the most commonly prescribed class of medications in the US and selective serotonin reuptake inhibitors (SSRIs) are the most commonly prescribed type of antidepressant. New evidence suggests that SSRI’s could inhibit the metabolic conversion of certain opioids known as prodrug opioids, e.g. hydrocodone and codeine decreasing their efficacy for pain management. Therefore understanding the effect of SSRI antidepressants on postoperative pain management is essential as the US strives to move to a precision health system, and as opioid misuse and addiction continues to rise in the US during the current epidemic [8].

Opioids are thought to exert their analgesic effects by binding to the Mu opioid receptor in the brain and spinal cord. Some opioids directly bind to the mu-opioid receptor in their native form including oxycodone, morphine, hydromorphone, fentanyl, and methadone [9]. Other opioids require chemical conversion to an active form by a de-methylation reaction mediated in the human liver by CYP-2D6, a member of the cytochrome p450 enzyme system [10,11]. Such drugs are known as prodrug opioids, requiring metabolism and chemical modification to exert their pharmacological effect. Examples include hydrocodone- the most commonly prescribed drug in the nation which is the opioid ingredient in Vicodin, Lortab, and Norco. A recent study suggested that the interaction of a CYP-2D6 inhibitor might be important in reducing the effectiveness of hydrocodone [12]. Understanding the effects on pain control of the common antidepressant SSRI, a CYP-2D6 inhibitor, is essential to manage pain control is this vulnerable population with mental illness.

Our study’s objective was to quantify the effect of the combined administration of prodrug opioids and SSRI antidepressant medication on postoperative pain among depressed patients undergoing surgical procedures. We hypothesized that patients taking SSRI who are prescribed a prodrug opioid will have worse postoperative pain. We further hypothesized that we could utilize a machine learning approach to predict the increase or decrease in the discharge, 3-week and 8-week postoperative pain scores stratified by prescription of a prodrug opioid.

## Methods and materials

### Study design

Using electronic health records (EHRs) from a tertiary care academic medical center, we identified patients undergoing a major operating room procedure from 2009 to 2016 based on ICD-9 codes. We built a predictive model for postoperative pain evaluation using ElasticNet regularized regression and internally cross-validated the model using 10-fold cross validation [13,14].

### Data source and patient population

This study and a waiver of written consent was approved by Stanford University’s Internal Review Board, approval #34551. This study used routinely collected medical data captured in electronic health records. Patients were identified from the EHR from Stanford University Hospitals and Clinical, an academic hospital that has maintained the EHR system (Epic Systems, Verona WI) since 2008. We identified patients undergoing common surgical procedures using ICD-9/10 and CPT codes, including orthopedics, vascular, and general surgeries. (S1 Table) Patients were excluded if age at surgery was less than 18 years or death occurred during the hospitalization. For patients with multiple surgeries, only first surgery was included. Patients with concurrent procedures were included in the analysis. For each patient in the cohort, we captured information regarding patient demographics, diagnosis, treatment plans, procedures, diagnostic results within 30 days prior to the date of surgery, other medications within 30 days prior to the date of surgery, and clinical notes (History & Physical, progress notes, and discharge summaries). Charlson score was calculated based on secondary diagnoses 12 months prior to surgery.

### Study variables

#### Patient characteristics

Patient variables included Gender, Race/ethnicity (White, Hispanic, Black, Asian & Other), age at surgery, marital status, insurance type (Private, Medicaid, Medicare and Other), Charlson Comorbidity Index (no comorbidity, Charlson score one, and a Charlson score two or more), and body mass index (BMI). Patient vital information included deviation of the systolic and diastolic blood pressure from the normal range of 80 mm/Hg and 120 mm/Hg respectively. The heart rate was represented as a categorical variable with values normal, abnormal, or unknown. Body temperature deviation from the normal value was also included. Preoperative pain was included as the latest reported mean pain score up to 30 days before date of surgery. Medications were represented as binary indicator for the presence or absence of a particular therapeutic class of medications within 30 days prior to the surgery. Opioid tolerant (non-naïve) patients were defined as any patient with an outpatient opioid prescription within 12-months prior to the admission for the surgical procedure.

#### Pain medications

The drug formulary and vocabularies were mapped to a 2016 version of RxNorm. Prescription orders were distilled to the ingredient level. The algorithm used for data extraction accounted for any combination medications and trade names. The average daily oral morphine consumption for the inpatient stay was calculated using oral morphine equivalent conversion factors [15].

#### Antidepressants and depression

Patients were included in the cohort if they had a diagnosis, medication, or symptom for depression within 1 year of the surgery date using a combination of structured and unstructured EHR data. The NLP algorithm (described below) identified clinical concepts from the unstructured clinical notes and medications and diagnosis codes were extracted from structured variables (S2 Table). Similarly, patients on SSRIs were identified from the clinical notes as well as from the structured medication records, SSRI+/- (S3 Table).

#### Opioid classes: Prodrugs and non-prodrugs

**Fig 1:**
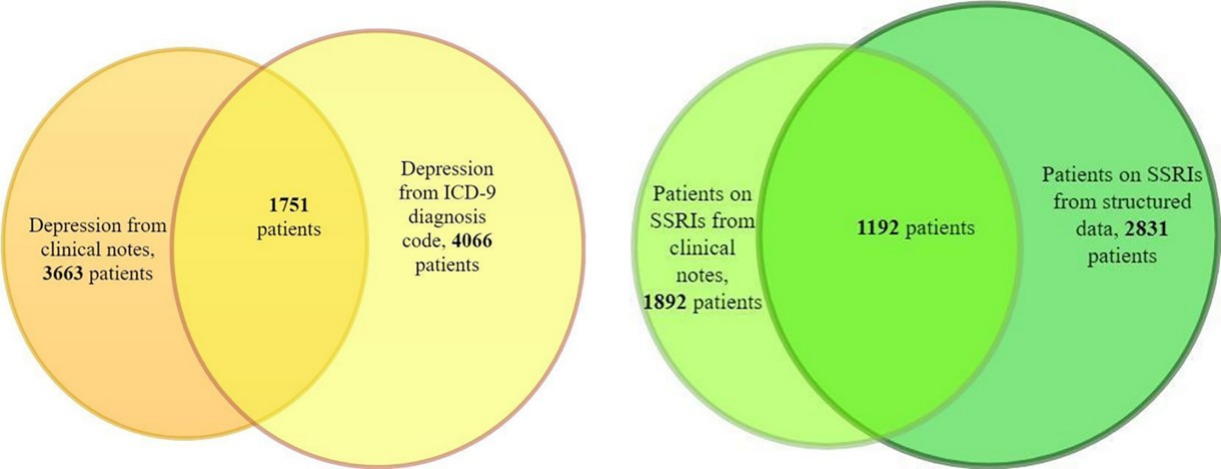
Identification of depressed patients and patients on SSRIs using clinical notes and structured data.

Opioids were classified as a prodrug based on previous literature review [10]. Prodrug opioids (Prodrug+) included: Hydrocodone, Codeine, and Tramadol. Non-Prodrug opioids (Prodrug-) included: Morphine, Fentanyl, Hydromorphone, Oxycodone, and Methadone. Fig 1 demonstrates how the different types of data were combined to create the final cohorts.

### Clinical outcomes

We assessed pain outcome measurements according to the numerical rating pain score [16]. Pain scores were routinely collected on a scale ranging from 0–10, where 0 indicates “no pain” and 10 indicates “worst pain”. Return visits differed between surgeries. We included 3 postoperative follow-ups for pain score assessment: discharge pain, mean pain score recorded on the last inpatient day; 3 week follow-up (+/- 7 days) and 8 week follow-up (+/- 7 days). To address inter-patient subjectivity in pain perception, we compute pain control as the difference between the postoperative and the preoperative pain score for each patient.

### Model development

We constructed and evaluated all prediction models with repeated 10-fold cross validation, which partitions the original sample into ten subsets, and uses nine of those subsets in the training process and then makes predictions on the remaining subset. We averaged model performance metrics across test folds.

#### NLP pipeline

The NLP algorithm works with unstructured clinical notes to identify depressed patients. A pre-processing step was performed that involved converting the numeric values to string, converting the text to the standard encoding format of UTF-8, normalizing case, and to remove any extraneous spaces, lines, or characters. Each cleaned note is tokenized, which means it was split into identifiable elements–in this case, words and punctuation, sentence boundary detection, identification all the stop words, and removal of ≤ 2 letter words using Natural Language Toolkit (NLTK) [17]. We incorporate a domain specific dictionary containing a curated list of relevant terms (e.g. depression) from the Unified Medical Language system (UMLS). The next step was to design a set of rules based on manual review of 300 randomly selected notes for filtering out irrelevant sentences (e.g. depression related to orthopedic conditions and electrocardiogram (ECG) depressions). Each sentence was then annotated as *Affirmed* or *Negated* using the NegEx algorithm, which identifies the negatives pertinent to the diagnosis in each sentence, see examples in Table 1 [18]. As each note is made up of multiple sentences, sentence level annotations were aggregated to the note level using a majority voting algorithm where the *Affirmed* label was given the priority in case of a tie. The performance of the NLP algorithm was evaluated using randomly sampled 100 manually annotated clinical notes using standard metrics: precision, recall and the F1 score.

**Table 1:** Sample sentences and the corresponding annotations for depression symptoms.

Text Snippet	Label
“major depressive disorder, recurrent episode, moderate”	Affirmed
“stress and depression, need to continue medication”	Affirmed
“patient stating more depression recently, was at behavioral facility and is now more paranoid”	Affirmed
“patient had no preinjury mental health issues or depression”	Negated
“anxiety was situational, currently no symptoms of depression”	Negated

#### Predictor selection

In order to predict the increase or decrease of three discrete postoperative follow up pain scores, we built three isolated classification models using an integrated feature vector which was constructed using a subset of EHR record data captured before surgery. Particularly, the feature vector was built upon six EHR components: patient demographics, clinical characteristics, diagnoses information, medications, vital statistics, and a categorical variable to indicate the patient group: SSRI+/- and Prodrug+/-. The final feature vector consisted of 65 variables.

#### Predictive model building

We used the 65 predictive features to train three individual machine-learning models to predict change in postoperative pain *(Δt = PostOpPain*_*t*_*—PreOpPain)* at three discrete time points (*t*): (1) at discharge, (2) 3 weeks, and (3) 8 weeks after surgery. We used an ElasticNet regularized regression, which combines L1 penalties of Lasso and L2 penalties of Ridge where the objective function is defined as:

$$\hat{B}={argmin}_{\beta }{\left|\left|{∆}_{t}-\beta X\right|\right|}^{2}+\alpha \lambda .{Ridge}_{Penalty}+\frac{\lambda }{2}(1-\alpha ).{Lasso}_{Penalty}$$

and *X* is the 65 dimensional feature matrix, *β* is the computed coefficients, λ controls the overall strength of the penalty, *α*ϵ[0,1]. ElasticNet performed shrinkage of the quantitative feature matrix while maintaining the pairwise correlation between features which allows to select more than one correlated variable. Given the possibility of high correlation between the EHR variables, selection of grouped features rather than sparse selection is an important aspect of the prediction. Due to the limited number of training samples, instead of multi-level regression, we formulated the prediction problem as binary where we predicted the improvement/worsen of the absolute pain score at discharge, 3 weeks and 8 weeks compared to the preoperative baseline pain score of each individual.

#### Model validation

Our regularized regression models are designed to predict change in postoperative pain outcomes among depressed patients who were either taking an SSRI or receiving an opioid prodrug. We use 10-fold cross validation where the original training sample at each time-point is randomly partitioned into 10 equal size subsamples. Of the 10 subsample sets, a single set is retained as the validation data for testing the model, and the remaining 9 subsamples are used as training data. The cross-validation process is then repeated 9 times (the folds), with each of the 10 subsamples used exactly once as the validation data. The main advantage of this method is that all patients included in our cohort are used for both training and validation, and each observation is used for validation exactly once. The hyper parameter tuning was performed using a 5-fold nested cross validation on the train set for each fold. The hyper parameters corresponding to the best model were chosen empirically to be alpha = 0.3, lambda = 0.05.

In order to test generalizability of the proposed model, we presented the receiver-operating curve (ROC) for each test fold and computed the mean ROC by combining the results of each fold. We consider the mean of the area under the receiver-operating curve (AUCROC) as the final validation metrics. All analyses and validation were implemented in python using scikit-learn library. All python-code we developed for statistical modelling is available upon request.

## Results

Among total of 41,713 patients, 4306 patients are identified with depression using clinical notes and structured variables. There were 1164 males and 3142 females in the study with a mean age of 58.34 years. The cohort consisted of 69.5% patients to be White, followed by Hispanic, Asian, Black and other races. There was no statistical difference in the receipt of a discharge prodrug opioid by SSRI prescription (p = 0.4196). Table 2 shows the patient demographics across the four groups of interest. There were 606 patients classified as SSRI+/Prodrug+, 1285 patients SSRI+/ Prodrug-, 802 patients SSRI-/Prodrug+ and 1613 SSRI-/Prodrug-. The clinical characteristics of the patients in the cohort is shown in Table 3. The mean preoperative pain score on a scale of 0–10 for the entire cohort was 2.62 with a standard deviation of 3.07.

**Table 2:** Baseline patient characteristics stratified by SSRI and prodrug opioid prescription, 2009–2017.

Characteristic		Total Cohort	SSRI+			SSRI-		
			Prodrug+	Prodrug-	p-value	Prodrug+	Prodrug-	p-value
**Total**, n (%)		4306 (100)	606 (14.07)	1285 (29.84)		802 (18.63)	1613 (37.46)	0.4196
**Age** in years, mean (SD)		58.34 (14.88)	57.68 (15.06)	59.49 (14.76)	0.0132	57.71 (15.23)	57.99 (14.67)	0.6680
**Gender**, n (%)	Male	1164	149 (24.59)	353 (27.47)	0.1852	208 (25.94)	454 (28.15)	0.2513
	Female	3142	457 (75.41)	932 (72.53)		594 (74.06)	1159 (71.85)	
**Race/ Ethnicity**, n (%)	White	2969	412 (67.99)	928 (72.22)	0.4177	523 (65.21)	1106 (68.57)	0.3848
	Black	134	16 (2.64)	32 (2.49)		31 (3.87)	55 (3.41)	
	Hispanic	570	79 (13.04)	151 (11.75)		118 (14.71)	222 (13.76)	
	Asian	254	34 (5.61)	62 (4.82)		62 (7.73)	96 (5.95)	
	Other	379	65 (10.73)	112 (8.72)		68 (8.48)	134 (8.31)	
**Marital Status**, n (%)	Married/ Life Partner	2420 (56.20)	348 (57.43)	723 (56.26)	0.6345	442 (55.11)	907 (56.23)	0.6022
	Single**	1886 (43.80)	258 (42.57)	562 (43.74)		360 (44.89)	706 (43.77)	
**BMI**, mean (SD)		28.47 (7.39)	28.16 (7.20)	28.97 (7.28)	0.0234	27.90 (6.90)	28.48 (7.75)	0.0702
**Insurance Type**, n (%)	Private	1372 (31.86)	184 (30.36)	363 (28.25)	0.1427	269 (33.54)	556 (34.47)	0.5974
	Medicaid	502 (11.66)	81 (13.37)	152 (11.83)		97 (12.09)	172 (10.66)	
	Medicare	2057 (47.77)	280 (46.20)	664 (51.67)		362 (45.14)	751 (46.56)	
	Other	375 (8.71)	61 (10.07)	106 (8.25)		74 (9.23)	134 (8.31)	
**Charlson Score,** n (%)	0–2	3775 (87.67)	532 (87.79)	1136 (88.40)	0.1502	690 (86.03)	1417 (87.85)	0.2082
	3+	531 (12.33)	74 (12.21)	149 (11.60)		112 (13.97)	196 (12.15)	

**Single/Widowed/ Divorced/ Separated

**Table 3:** Clinical characteristics stratified by SSRI use and prodrug prescription, 2009–2017.

Characteristic		Total Cohort	SSRI+			SSRI-		
			Prodrug+	Prodrug-	p-value	Prodrug+	Prodrug-	p-value
**Opioid Naive**, n(%)	Yes	1836 (42.64)	282 (46.53)	566 (46.05)	0.3100	343 (42.77)	645 (39.99)	0.1906
	No	2470 (57.36)	324 (53.47)	719 (55.95)		459 (57.23)	968 (60.01)	
**Daily OME, mean (SD)**		61.91 (56.96)	61.52 (56.21)	63.94 (54.10)	0.1634	56.60 (62.43)	63.14 (56.49)	0.01431
**Pre-operative pain**, n		3326	472	994		615	1245	
Pre-operative pain*, mean (SD)		2.62 (3.07)	2.29 (2.92)	2.93 (3.22)	0.0003	2.22 (2.88)	2.70 (3.05)	0.0009
**Discharge Pain, n**		4189	596	1239		791	1563	
**Discharge Pain,** mean (SD)		2.99 (2.24)	2.96 (2.07)	3.07 (2.32)	0.3718	2.66 (2.01)	3.12 (2.32)	< .0001
Postoperative 3 week Pain*, n		4030	568	1221		737	1504	
Postoperative 3 week Pain*, mean (SD)		4.58 (2.39)	4.76 (2.40)	4.56 (2.33)	0.1028	4.29 (2.42)	4.68 (2.40)	0.0003
**Postoperative 8 week pain, n**		2682	379	841		458	1004	
Postoperative 8 week Pain*, mean (SD)		3.11 (3.01)	2.99 (3.18)	3.21 (2.99)	0.2434	2.84 (2.84)	3.19 (3.02)	0.0368

*Numeric rating score, scale 0–10

The developed NLP algorithm achieved precision of 0.92, recall of 0.987 and an F1 score of 0.95 when being compared against gold standard of 100 manually annotated clinical notes. A total of 3663 patients were identified as depressed using clinical notes while 4066 patients were identified to be depressed from the ICD-9 diagnosis codes. 1751 patients were identified as depressed in both methods. The NLP algorithm helped to identify an additional 1912 depressed patients who were not recorded for depression using ICD-9 codes at the academic hospital but their notes indicated that they were depressed patients. Similarly, an additional 700 patients were identified to be on SSRIs apart from those patients that were obtained from the structured medication data. Fig 1 shows the overlap of patients identified from the structured and the unstructured data for depression diagnosis and SSRI medication.

Pain control, as measured by the mean difference between the postoperative and preoperative pain scores, are shown in Table 4. Lower mean difference in pain scores indicated better pain control. Among the SSRI+ patients, those in the Prodrug+ group had a mean difference in pain scores at discharge of 0.720 in comparison to 0.161 for SSRI+/Prodrug- group (p = 0.0022). This trend was similar at both 3 weeks and 8 weeks postoperative (p < .0001 and p = 0.0133, respectively). When comparing patients in the SSRI- groups, there was no difference in the mean difference pain scores across all postoperative time points (p>0.05).

**Table 4:** Mean change in pain score, stratified by SSRI medication and opioid prodrug prescription*.

Time Period	Total Cohort	SSRI+			SSRI-		
		Prodrug+	Prodrug-	P-value	Prodrug+	Prodrug-	P-Value
**Discharge Pain, mean (SD),** **(min, max)**	0.434	0.720 (3.15), (-8.57, 8.16)	0.161 (3.29), (-10, 10)	0.0022	0.492 (3.16), (-10, 8.2)	0.511 (3.20), (-10, 8.87)	0.9060
**3 weeks Postoperative, mean (SD),** **(min, max)**	2.080	2.774 (3.22), (-6.10, 10)	1.658 (3.38), (-9, 10)	< .0001	2.138 (3.18), (-10, 9)	2.126 (3.17), (-7, 10)	0.9430
**8 weeks Postoperative, mean (SD),** **(min, max)**	0.604	0.861 (3.89), (-9, 10)	0.215 (3.89), (-10, 10)	0.0133	0.798 (3.45), (-9, 10)	0.744 (3.44), (-10, 10)	0.7939

*Mean Change in Pain Score is the difference in the postoperative pain score minus the preoperative pain score, smaller values are greater improvement

**Fig 2:**
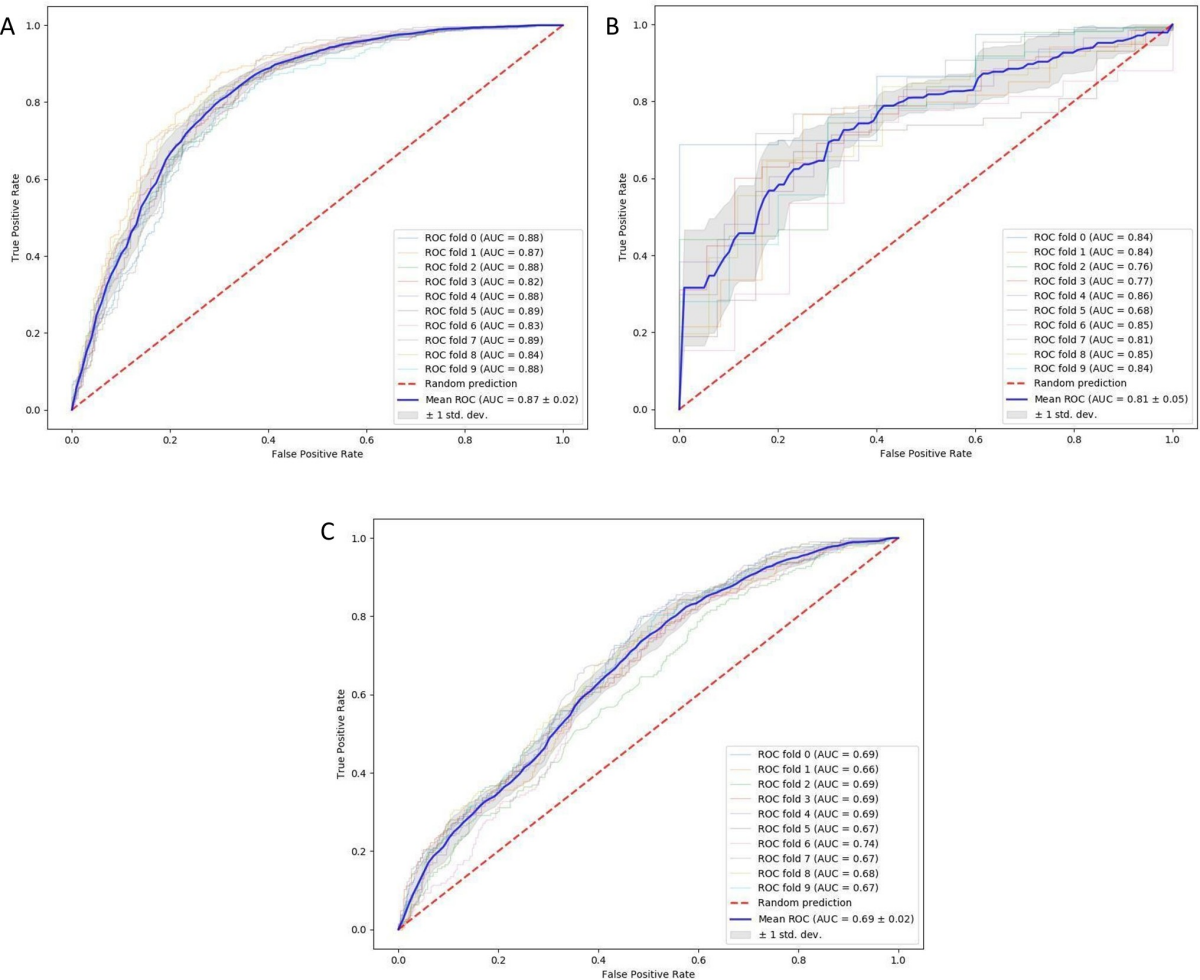
Receiver operator characteristic (ROC) curves for the model’s performance with 10-fold cross validation at three postoperative time-points. (A) ROC-AUC for prediction of pain score change at discharge. (B) ROC-AUC for prediction of pain score change at 3 weeks. (C) ROC-AUC for prediction of pain score change at 8 weeks.

We used support vector machine classifiers, random forest classifiers, random forest regressor and ElasticNet regularization models each with a 10-fold cross validation for predicting the change in pain scores at three distinct time points. The performance of each of these models are shown in Table 5. We found that, the ElasticNet regularization models with a 10-fold cross validation provided the best performance. For predicting alteration of pain at discharge (Δ_discharge_), the ElasticNet model with a 65-dimensional feature vector achieved a mean AUC of 0.87 with +/- 0.02 standard deviation, while the model achieved mean AUC of 0.81 +/- 0.05 and 0.69 +/- 0.02 for 3 weeks (Δ_3week_) and 8 weeks(Δ_8week_) respectively. The ROC indicating the model’s performance at each fold are shown. (Fig 2)

**Table 5:** Comparison of the performance of the models using area under the curve (AUC) of ROC.

	Classifier		Regressor	
Model	Support Vector Classifier	Random Forest Classifier	Random Forest regressor	Elastic Net
**Change in pain at Discharge,** **mean (SD)**	0.58 (+/-0.08)	0.81 (+/-0.02)	0.82 (+/-0.02)	0.87 (+/-0.02)
**Change in pain at 3 weeks Postoperative, mean (SD)**	0.53 (+/-0.11)	0.82 (+/-0.07)	0.75 (+/-0.03)	0.81 (+/-0.05)
**Change in pain at 8 weeks Postoperative, mean (SD)**	0.52 (+/-0.08)	0.66 (0+/-.01)	0.66 (+/-0.03)	0.69 (+/-0.02)

**Fig 3:**
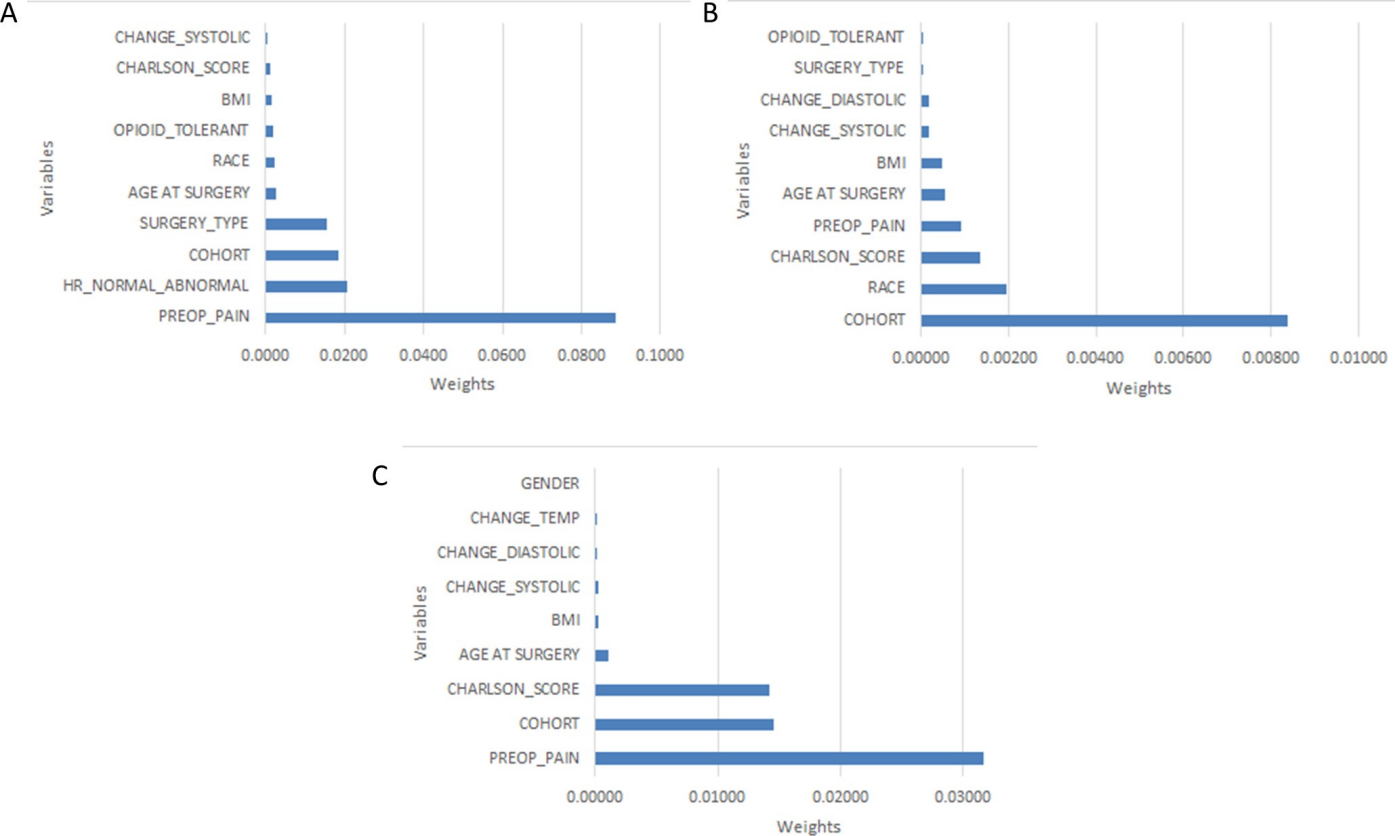
Discriminative features selected by the three models: Coefficients computed by the ElasticNet models are represented as weights. (A) discharge postoperative pain score predictions. (B) 3-week postoperative pain score predictions. (C) 8-week postoperative pain score predictions.

We represented the feature coefficient values (non-zero weights only) where the ranking of the features is derived by analyzing the coefficient of the features (*β*) computed by the 10-fold cross validated ElasticNet (Fig 3). Among 65 dimensional feature vector, preoperative pain is ranked highest for predicting pain at discharge, while the combination of SSRI and prodrug opioid exposure (named as COHORT), is ranked highest for 3 weeks pain prediction. Race is second discriminative feature for 3 weeks’ prediction. Similarly, for 8 weeks’ pain score prediction, SSRI and prodrug opioid exposure and preoperative pain are the most discriminative features.

## Discussion

The findings from this study demonstrate that depressed patients receiving SSRIs and prodrug opioids had inferior postoperative pain control and this drug-drug combination can accurately predict the increase or decrease in pain scores at discharge, 3 week and 8 week follow-up visits. Using real-world data to leverage a diverse population of surgical patients this study had the unique power to generate robust evidence on this drug-drug combination. We found that although SSRI patients on non-prodrug opioids had higher preoperative pain, a significant risk factor for worse post-operative pain, these patients fared *better* in regard to post-operative pain control compared to patients receiving prodrug opioids. We also present evidence that this drug-drug interaction is not taken into account when prescribing analgesics; the prescribing of prodrug opioids did not differ by SSRI medications. Our findings indicate that depressed patients taking SSRIs should receive a direct-acting rather than prodrug opioid for postoperative pain control.

In this study, we observed that patients receiving a non-prodrug opioid had higher preoperative pain scores. Higher preoperative pain scores can likely bias opioid prescribing, as clinicians may likely give these patients a non-prodrug, such as oxycodone, which can be given more frequently, or a hydromorphone which is more potent. Given the fact that higher preoperative pain is one of the greatest risk factors for worse postoperative pain outcomes, clinicians may appropriately prescribe stronger acting non-prodrug opioids to this group which may be at higher risk for postoperative pain [19, 20]. Interestingly, in the non-SSRI group, patients receiving non-prodrug opioids had *better* pain outcomes compared to the prodrug opioid group. It is therefore likely that the effect of the prodrug-SSRI interaction on pain may be larger than we estimate given this selection bias. Further understanding of these results require future validation in other healthcare settings.

Patients with psychiatric disorders represent a vulnerable population and report greater levels of pain, less benefit from treatment, and higher use of opioids postoperatively. SSRIs are both the most commonly prescribed class of psychiatric medications and inhibit the action of prodrug opioids, the most common pain medication prescribed. This suboptimal drug/drug combination may mediate a portion of the adverse pain outcomes seen in psychiatric patients. This study suggests that clinicians should account for the SSRI-Prodrug interaction and instead consider an active form of opioids (e.g. oxycodone or fentanyl) when prescribing to this population. Indiscriminate opioid prescriptions have contributed to the current opioid epidemic [21]. Identification of specific situations- such as SSRI use- in which specific opioid prescriptions are less effective, may be one small part of a larger effort to develop a more discriminating, personalized, educated opioid prescribing.

This study of real-world evidence revealed that depressed patients taking the combined SSRI and Prodrug opioid combination had worse postoperative pain control compared to patients in the SSRI non-Prodrug opioid group, while depressed patients not taking an SSRI had no difference in pain control by Prodrug opioid use. These differences were statistically significant and approach the threshold of minimal clinically significant difference (MCSD) in pain scores [22]. Prodrug opioids are demethylated by CYP-2D6 in the human liver to become active, which is what creates the analgesic activity. Many studies have demonstrated how the inhibition of CYP-2D6 has effects on opioid pain modulating effects and the potential for inhibition by SSRI’s to meaningfully reduce opioids effectiveness [23–25]. This study is the first to our knowledge that tests these hypotheses in the uncontrolled clinical setting however further research is needed to understand the MCSD in vulnerable populations.

The success of machine learning models in medicine depends not only on accuracy of prediction but also their ability to generalize. In this study, the algorithms had high accuracy, which shows a good prediction ability of the model as well as little deviation in AUCs between folds in 10-fold cross validation for both discharge pain and 3-week postoperative pain, which shows less overfitting during training thus good ability to generalize. Moderate prediction performance at 8 weeks mainly shows complexity of the task, in which the prediction of 8 weeks follow up pain change is done by only looking at the preoperative data. Crucially, this study offers a pipeline to identify useful predictors for poor pain management and to validate clinical trials or research hypotheses using real world evidence.

If replicated, our results have multiple clinical implications. First, clinicians prescribing prodrug opioids should routinely screen for SSRI use and consider it in their decision making. To ensure consistent, predictable effect of prescribed opioids, it may be advisable where possible to prescribe opioids whose effect does not depend in liver metabolism (e.g., oxycodone). In cases where this is not possible, patients should be warned that any change in their SSRI use could have implications for their pain level and opioid-related risk. For example, a patient on high-dose hydrocodone who stops taking SSRIs may begin experiencing an effectively higher opioid dose, raising their risk of adverse side-effects (e.g., constipation, somnolence, overdose). For their part, clinicians initiating patients onto SSRIs should inquire about any prodrug opioids the patient is taking, as they may wane in effectiveness when the psychiatric medication is taken. This is worrisome both because it could increase pain but also because it could lead patients to ramp up their opioid dose on their own, exposing themselves to risk.

There are limitations to this study. First, we use data from a single academic medical institute. While these results might have limited generalizability to other populations, because we use all patients seeking treatment at this tertiary care academic center and include a wide variety of surgical procedures, the results are likely reproducible in other population cohorts. Second, we capture only medication that was prescribed or documented in notes, as we do not have information in medications ingested outside of the inpatient setting. However, this included notes from visits 6 months prior to surgery, the inpatient stay, and postoperative/follow-up visits through 90 days’ discharge. Furthermore, at 8 weeks’ follow-up we have a significant number of patients lost to follow-up, a common limitation at tertiary academic centers. Third, two drugs examined have a complex relationship between their metabolism and activity. Oxycodone is active and potent at the mu-opioid receptor in its native form, but is to some extent metabolized to yet a more potent form [25]. Similarly tramadol is demethylated to its active opioid metabolite, but the parent compound has some non-opioid analgesic activity [26]. These complex metabolism activities could bias our results toward the null causing us to *underestimate* the true analgesic difference between direct acting opioid and prodrug opioids in the SSRI consuming patient. However, because of their common use we include these complicated analgesics in the most conservative way guided by existing evidence of their effects. Additionally, our findings are based on retrospective EHRs, which include inherent bias. However, substantial efforts were made to check accuracy of selection and extractions of variables based on completeness, clinical meaningfulness and distributions. Finally, these results are predictive and do not suggest causality.

In conclusion, our results suggest that prodrug opioids for postoperative pain control after discharge are less effective than active form opioids in patients on SSRIs. The inhibition of metabolic conversion of the prodrug opioids by the SSRI had been theorized to impair pain control, however ours is the first study to demonstrate this interaction in a clinical population of depressed patients using SSRI’s therapeutically. Our predictive model may help future researchers better personalize pain management regimes. If confirmed, prescribers should consider alternatives to prodrug opiates in pain management for patients taking SSRIs.

## Supporting information

S1 Table ICD-9, ICD-10 and CPT codes used for identifying the surgical procedures included in the study. (PDF)

S2 Table Unified medical language system’s depression terms used to identify key concepts from clinical notes for depression diagnoses and symptoms. (PDF)

S3 Table List of drugs included in the SSRI drug class and their trade names. (PDF)
