# Supplementary material for: Predicting inadequate postoperative pain management in depressed patients: A machine learning approach
Source: PLoS One. 2019 Feb 6;14(2):e0210575. doi: 10.1371/journal.pone.0210575 (PMC6364959; doi:10.1371/journal.pone.0210575)
Supplement: S1 Table — (PDF) [file pone.0210575.s001.pdf]

S1 Table. ICD-9, ICD-10 and CPT codes used for identifying the surgical procedures included in the study

Classification	ICD-9	ICD-10 and CPT
Orthopedics (Hip Replacement, Knee Replacement, Distal Radius Fracture	81.51, 81.52, 81.53, 81.54,	0SRC0J9, 0SRC0JA, 0SRC0JZ, 0SRC0KZ, 0SRD0J9, 0SRD0JA, 0SRD0JZ, 0SRT0J9, 0SRU0J9, 0SRU0JA, 0SRU0JZ, 0SRV0J9, 0SRV0JA, 0SRV0JZ, 0SRW0J9, 0SRW0JZ, 25606, 25607, 25608, 25609, 27125, 27130, 27132, 27134, 27137, 27138, 27437, 27438, 27440, 27442, 27443, 27445, 27446, 27447
General (Appendectomy, Cholysectomy, Hernia)	17.11, 17.12, 17.13, 17.21, 17.22, 17.23, 17.24, 51.21, 51.22, 51.23, 51.24, 53.01, 53.02, 53.03, 53.04, 53.05, 53.11, 53.12, 53.14, 53.15, 53.17, 68.51	44950, 44955, 44960, 44970, 47.01, 47.09, 47.11, 47.19, 47562, 47563, 47564, 47579, 47600, 47605, 47610, 49500, 49505, 49507, 49520, 49521, 49525, 49650, 49651, 58150, 58180, 58275, 58290, 58291, 58292, 58294, 58541, 58542, 58543, 58544, 58545, 58546, 58550, 58552,

		58553, 58554,<br>58570, 58571,<br>58572, 58573,<br>58578
Breast (Mastectomy, Lumpectomy, Excision)	85.33,<br>85.34,85.35,<br>85.36,<br>85.41, 85.42,<br>85.43, 85.44,<br>85.45, 85.46,<br>85.47, 85.48	0HTT0ZZ, 0HTU0ZZ, 0HTV0ZZ, 07T50ZZ, 07T60ZZ, 0KTH0ZZ, 0KTJ0ZZ, 07T70ZZ, 07T80ZZ, 07T90ZZ,<br>19125, 38745<br>19126, 19301,<br>19302, 19303,<br>19305, 19306,<br>19307, 38500,<br>38525, 38740,
Vascular (Abdominal aortic Aneurysm, Amputation)	38.44, 39.71,<br>84.15, 84.17	
Breast Reconstruction		19316, 19318,<br>19324, 19325,<br>19340, 19342,<br>19350, 19357,<br>19361, 19364,<br>19367, 19368,<br>19369, 19370, 19371

Thoracotomy	34, 32.39, 32.49, 32.59	0W9800Z, 0W980ZZ, 0W9830Z, 0W983ZZ, 0W9840Z, 0W984ZZ, 0WP80JZ, 0WP83JZ, 0WP84JZ, 02JA0ZZ, 0WJC0ZZ, 0W390ZZ, 0W3B0ZZ, 0W3D0ZZ, 0W3Q0ZZ, 0W9930Z, 0W9940Z, 0W9B30Z, 0W9B40Z, 0W190JG, 0W194J4, 0W1B0JG, 0W1B4JG, 0B9N40Z, 0B9N4ZZ, 0B9P40Z, 0B9P4ZZ, 0B9N00Z, 0B9N0ZZ, 0B9N30Z, 0B9P00Z, 0B9P0ZZ, 0B9P30Z, 0W9900Z, 0W990ZZ, 0W994ZZ, 0W9B00Z, 0W9B0ZZ, 0W9B4ZZ, 0WC90ZZ, 0WC93ZZ, 0WC94ZZ, 0WCB0ZZ, 0WCB3ZZ, 0WCB4ZZ, 0BBK0ZZ, 0BBK3ZZ, 0BBK7ZZ, 0BBL0ZZ, 0BBL3ZZ, 0BBL7ZZ, 0BTC0ZZ, 0BTD0ZZ, 0BTF0ZZ, 0BTG0ZZ, 0BTJ0ZZ, 0BTK0ZZ, 0BTL0ZZ, 0BTM0ZZ, 0BDN0ZX, 0BDN0ZZ, 0BDN3ZX, 0BDN3ZZ, 0BDP0ZX, 0BDP0ZZ, 0BDP3ZX, 0BDP3ZZ
