# Supplementary material for: Predicting inadequate postoperative pain management in depressed patients: A machine learning approach
Source: PLoS One. 2019 Feb 6;14(2):e0210575. doi: 10.1371/journal.pone.0210575 (PMC6364959; doi:10.1371/journal.pone.0210575)
Supplement: S2 Table — (PDF) [file pone.0210575.s002.pdf]

S2 Table. Unified Medical Language system's depression terms used to identify key concepts from clinical notes for depression diagnoses and symptoms.

Depression Terms		
Adjustment disorder with depressed mood	Chronic recurrent major depressive disorder	Schizoaffective disorder, depressive type
Brief depressive adjustment reaction	Recurrent major depressive disorder with catatonic features	Post-schizophrenic depression
Mental Depression	Recurrent major depressive disorder with atypical features	Bipolar affective disorder, currently depressed, mild
Depression, Chemical	Recurrent major depressive disorder with postpartum onset	Bipolar affective disorder, currently depressed, moderate
Endogenous depression	Minor depressive disorder	Bipolar affective disorder, currently depressed, severe, with psychosis
Involutional Depression	Endogenous depression - recurrent	Bipolar affective disorder, currently depressed, in full remission
Reactive depression	Postviral depression	Manic-depressive - now manic
Depressive disorder	Chronic depression	Single major depressive episode, severe, with psychosis
Single major depressive episode	Mild depression	Major depressive affective disorder, single episode, in partial or unspecified remission
melancholic depression	Moderate depression	Recurrent major depressive episodes, severe, with psychosis
Unipolar Depression	Severe depression	Senile dementia with depressive or paranoid features
seasonal depression	emotional and psychiatric depression	Chronic bipolar I disorder, most recent episode depressed
Depressive Symptoms	AODR depressive state	Severe depressed bipolar I disorder with psychotic features, mood-congruent
Depressive Syndrome	Recurrent major depressive disorder with melancholic features	Severe depressed bipolar I disorder with psychotic features, mood-incongruent
Arteriosclerotic dementia with depression	depression anxiety disorder	Bipolar I disorder, most recent episode depressed with catatonic features
Mild major depression, single episode	Cancer patients and suicide and depression	
Moderate major depression, single episode	Monopolar depression, single episode or unspecified	
Severe major depression, single episode, without psychotic features	Depressive character or personality	
Single major depressive episode, in full remission	Hyposomnia, insomnia or sleeplessness associated with depression	
Recurrent major depressive episodes	Hyposomnia, insomnia or sleeplessness associated with major depression	
Mild recurrent major depression	Hyposomnia, insomnia or sleeplessness associated with minor depression	
Moderate recurrent major depression		
Severe recurrent major depression without psychotic features		
Recurrent major depression in complete remission		
Atypical depressive disorder		

Antidepressant type abuse<br>Antidepressant type abuse, continuous<br>Antidepressant type abuse, episodic<br>Antidepressant type abuse, in remission<br>Prolonged depressive adjustment reaction<br>Adjustment disorder with mixed anxiety and depressed mood<br>Cerebral depression, coma, and other abnormal cerebral signs in fetus or newborn<br>Depression, Postpartum<br>Recurrent depression<br>Lifelong depressive personality trait<br>Depressed reaction<br>Agitated depression<br>Depression aggravated<br>Primary degenerative dementia of the Alzheimer type, senile onset, with depression<br>Multi-infarct dementia with depression<br>Major depression single episode, in partial remission<br>Recurrent major depression in partial remission<br>Organic mood disorder of depressed type<br>Mild major depression<br>Moderate major depression<br>Severe major depression without psychotic features<br>Major depression in remission	Hypersomnia associated with depression<br>Hypersomnia associated with major depression<br>Hypersomnia associated with minor depression<br>Adjustment reaction with anxiety and depression<br>Depressive disorder, NEC in ICD9CM_2014<br>Depressive disorder, NEC in MDR<br>Depressive disorder, NEC in CCS2003<br>winter depression<br>Major Depressive Disorder<br>Major depression, melancholic type<br>Self-control behavior: depression<br>Acute depression<br>Depression and Suicide Cancer Patient<br>Depression and Suicide<br>Depressed - symptom<br>Drug-induced central nervous system depression<br>Parkinsonism with alveolar hypoventilation and mental depression<br>Depressive Disorder, Treatment-Resistant<br>Severe recurrent major depression<br>clinical depression<br>Childhood Depression<br>Severe major depression<br>Recurrent major depressive episodes, mild<br>Chronic depressive personality disorder<br>Recurrent major depressive episodes, in full remission	Bipolar I disorder, most recent episode depressed with melancholic features<br>Bipolar I disorder, most recent episode depressed with atypical features<br>Bipolar I disorder, most recent episode depressed with postpartum onset<br>Depressed bipolar I disorder in remission<br>Bipolar II disorder, most recent episode major depressive<br>Mild bipolar II disorder, most recent episode major depressive<br>Moderate bipolar II disorder, most recent episode major depressive<br>Chronic bipolar II disorder, most recent episode major depressive<br>Severe bipolar II disorder, most recent episode major depressive without psychotic features<br>Bipolar II disorder, most recent episode major depressive with catatonic features<br>Bipolar II disorder, most recent episode major depressive with melancholic features<br>Bipolar II disorder, most recent episode major depressive with atypical features<br>Bipolar II disorder, most recent episode major depressive with postpartum onset<br>Severe bipolar II disorder, most recent episode major depressive, in remission

Major depression in partial remission<br>Major depression in complete remission<br>Major depression, single episode, in complete remission<br>Recurrent major depression in remission<br>Stuporous depression<br>Depression, Neurotic<br>depressive personality<br>Presenile dementia with depression<br>Senile dementia with depression<br>Drug-induced depressive state<br>Recurrent major depressive episodes, moderate<br>Major depressive affective disorder, recurrent episode, in partial or unspecified remission<br>Endogenous depression first episode<br>Masked depression<br>Mixed anxiety and depressive disorder<br>Brief depressive reaction<br>Depressive conduct disorder<br>Mild postnatal depression<br>Severe postnatal depression<br>Pseudo-Cushing's syndrome of depression<br>Depressive episode, unspecified<br>Postoperative depression<br>Feeling depressed<br>Mood disorder with depressive features due to general medical condition	Adjustment disorder with depressed mood in remission<br>Depressive disorder in remission<br>Depressive disorder in mother complicating childbirth<br>Major depressive disorder in mother<br>Manic-Depression<br>Depression, Bipolar<br>Depression, Reactive, Psychotic<br>MANIC DEPRESSIVE DISEASE, MANIC PHASE<br>Severe major depression, single episode, with psychotic features<br>Severe recurrent major depression with psychotic features<br>Bipolar I disorder, most recent episode (or current) depressed<br>Depressed bipolar I disorder<br>Mild depressed bipolar I disorder<br>Moderate depressed bipolar I disorder<br>Severe depressed bipolar I disorder without psychotic features<br>Depressed bipolar I disorder in partial remission<br>Depressed bipolar I disorder in full remission<br>Manic Type Manic Depressive Illness<br>Severe major depression with psychotic features	Severe bipolar II disorder, most recent episode major depressive, in partial remission<br>Severe bipolar II disorder, most recent episode major depressive, in full remission<br>depressive delusion<br>depressive schizoaffective disorders<br>Depression, psychotic<br>depression psychotic feature<br>Senile dementia with delusional or depressive features<br>Manic-depressive psychosis, circular type but currently manic<br>Manic-depressive psychosis, circular type but currently depressed<br>Manic-depressive psychosis, circular type, mixed<br>Transient organic psychotic condition, depressive type<br>Manic-depressive psychosis or reaction, hypomanic, single episode or unspecified<br>Manic-depressive psychosis or reaction, manic, single episode or unspecified<br>Depressive psychosis, single episode or unspecified<br>Endogenous depression, single episode or unspecified<br>Manic-depressive psychosis or reaction, depressed type, single episode or unspecified<br>Psychotic depression, single episode or unspecified<br>Manic-depressive psychosis, circular type, current

Mood disorder with major depressive-like episode due to general medical condition<br>Menopausal depression<br>Chronic major depressive disorder, single episode<br>Major depressive disorder, single episode with catatonic features<br>Major depressive disorder, single episode with melancholic features<br>Major depressive disorder, single episode with atypical features<br>Major depressive disorder, single episode with postpartum onset	Severe major depression with psychotic features, mood-congruent<br>Severe major depression with psychotic features, mood-incongruent<br>Severe major depression, single episode, with psychotic features, mood-congruent<br>Severe major depression, single episode, with psychotic features, mood-incongruent<br>Severe recurrent major depression with psychotic features, mood-congruent<br>Severe recurrent major depression with psychotic features, mood-incongruent	condition not specified as either manic or depressive<br>Manic-depressive psychosis, mixed type<br>bipolar depression<br>depressed phase<br>Bipolar I disorder, most recent episode (or current) depressed<br>Manic-Depressive Illness<br>MANIC-DEPRESSIVE PSYCHOSIS<br>Reactive depressive psychosis, single episode<br>Severe depressed bipolar I disorder<br>Depressive type psychosis<br>Manic-depressive illness in children<br>Manic-depressive illness in adolescence
