# Supplementary material for: Predicting inadequate postoperative pain management in depressed patients: A machine learning approach
Source: PLoS One. 2019 Feb 6;14(2):e0210575. doi: 10.1371/journal.pone.0210575 (PMC6364959; doi:10.1371/journal.pone.0210575)
Supplement: S3 Table — (PDF) [file pone.0210575.s003.pdf]

S3 Table. List of drugs included in the SSRI drug class and their trade names

Drug	Trade name
Citalopram	Celexa, Cipramil
Escitalopram	Lexapro, Cipralex
Paroxetine	Paxil, Seroxat
Fluoxetine	Prozac
Fluvoxamine	Luvox, Faverin
Sertraline	Zoloft, Lustral
